# The complete mitochondrial genomes of the nematode-trapping fungus *Arthrobotrys oligospora*

**DOI:** 10.1080/23802359.2018.1507651

**Published:** 2018-08-29

**Authors:** Lili Jiang, Yunrun Zhang, Jianping Xu, Ke-Qin Zhang, Ying Zhang

**Affiliations:** a State Key Laboratory for Conservation and Utilization of Bio-Resources in Yunnan, and Key Laboratory for Southwest Microbial Diversity of the Ministry of Education, Yunnan University, Kunming, P. R. China;; b School of Life Science, Yunnan University, Kunming, P. R. China;; c Department of Biology, Mc Master University, Hamilton, Canada

**Keywords:** *Arthrobotrys oligospora*, mitochondrial genomes, phylogenetic relationships

## Abstract

*Arthrobotrys oligospora* is a potential candidate of biocontrol agents against plant and animal parasitic nematodes. In this study, the complete mitochondrial genome of *A.oligospora* was sequenced. This mitogenome is a circular molecule of 160,613 bp in length. Gene annotation showed that 44 putative protein-coding genes and 24 tRNAs, all located on the same strand. The evolutionary relationships between *A. oligospora* and other representative ascomycetes were revealed based on sequences at the 14 concatenated mitochondrial protein-coding genes.

*Arthrobotrys oligospora* Fresen, is one of the most extensively distributed and studied member in the attracting carnivorous fungi that can trap and infect nematodes using special hyphae forming a three-dimensional adhesive net. This species has become a potential biocontrol candidate of plant and animal parasitic nematodes, the relationships among populations of this predacious fungus, nematodes and other saprophytic competitions are of vital importance when applying biocontrol strains. While the nuclear genetic diversity and phylogenetic relationships with other carnivorous fungi have been investigated (Yang et al. 2007, Zhang et al. 2013, 2011, Zhang and Hyde 2014), little is known about its mitochondrial genome. Here, we report the complete mitogenome of *A. oligospora* and investigate its phylogenetic relationships with other ascomycetous fungi based on mitochondrial protein-coding genes.

The mitogenome was extracted from the whole genome sequence of a pure culture of strain American Type Culture Collection ATCC24927 (collected from Soil, Sweden, by B. Nordbring-Hertz, 1850). The mitogenome was identified by BLAST as scaffold 00048 (GenBank: JH119047) in whole genome, annotated using MFannot (http://megasun.bch.umontreal.ca/cgi-bin/mfannot/mfannotInterface.pl) and GLIMMER (https://www.ncbi.nlm.nih.gov/genomes/MICROBES/glimmer_3.cgi). The tRNAs were annotated using tRNAscan-SE (Schattner et al. 2005). All ORFs were searched and identified by ORFFinder.

The complete mitogenome sequence consists of 160,613 bp in length. There are 17 genes with 33 introns, 24 tRNAs and 37 ORFs were annotated. Those genes including rps3, ATP6 (1 group B intron), ATP8, ATP9, NAD1 (2 group A introns), NAD2 (4 group A introns), NAD3, NAD4 (2 group A introns), NAD4L, NAD5 (2 group A introns), NAD6, COB(3 group A introns), COX1 (12 group A introns), COX2 (7 group A intron), COX3, rnl and rns. The 24 tRNAs covered 16 standard amino acids without threonine, aspartate, cysteine, serine, tryptophan and lysine. In addition, there are 11 repeats is 3189 bp, 42 tandem repeats sequences is 939 bp and 33 palindromic sequences is 1635bp.

Phylogenetic analysis in 19 ascomycetes based on concatenated sequences of 14 conserved mitochondrial protein-coding genes was performed by Bayesian inference (BI). The best substitution model was analysed by prottest 3.0 (Best model according to AIC: LG + G + F). As shown in Figure 1. *A. oligospora* belong to Orbiliomycetes closely related to *Pyronema omphalodes*. *Candida* which belong to Saccharomycetes was taken as outgroup. The inferred relationship using mitochondrial genes is the same as the nuclear genes (Sugiyama et al. 2006). The Genbank Accession number for Arthrobotrys Oligospora is MK571436.

**Figure 1.**
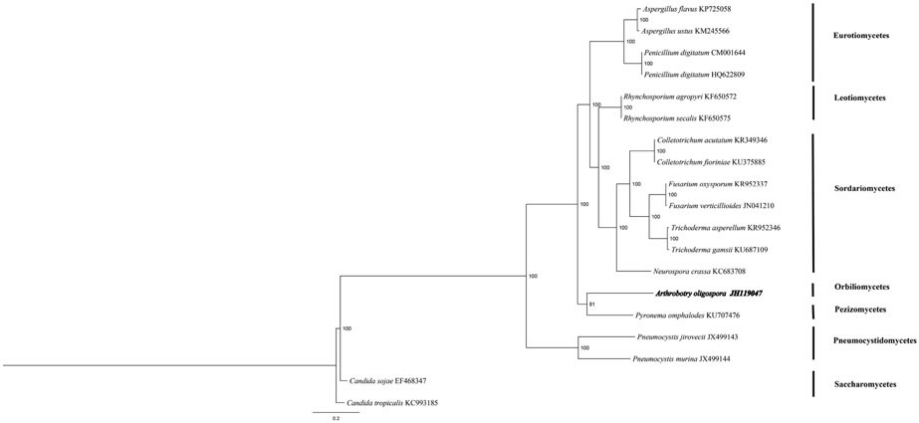
Phylogenetic relationships among 19 ascomycetes based on 14 conserved mitochondrial protein-coding genes. Phylogenetic analysis of 19 species of Agaricomycotina constructed using the Bayesian inference method as implemented in MrBayes based on concatenated amino acid sequences of 14 mitochondrial protein-coding genes. The following 14 mitochondrial protein-coding genes were concatenated: atp6, atp8, atp9, cytb, cox1, cox2, cox3, nad1, nad2, nad3, nad4, nad4L, nad5 and nad6. The concatenated amino acid sequences were aligned using Clustal X. The percentages of replicate trees in which the associated taxa clustered together in the bootstrap test (1,000,000 replicates) were shown next to the branches.
